# Comparison of Split Thickness Skin Grafts and Flaps in Bilateral Chronic Axillary Hidradenitis Suppurativa

**DOI:** 10.29252/wjps.9.1.55

**Published:** 2020-01

**Authors:** Abolfazl Afsharfard, Mohammad Bashir Khodaparast, Sina Zarrintan, Negin Yavari

**Affiliations:** 1 Department of General and Vascular Surgery, Shohada-Tajrish Medical Center, Shahid Beheshti University of Medical Sciences, Tehran, Iran;; 2 Research Department, Tehran Heart Center, Tehran University of Medical Sciences, Tehran, Iran

**Keywords:** Split thickness skin, Graft, Flap, Axillary, Hidradenitis suppurativa

## Abstract

**BACKGROUND:**

Hidradenitis suppurativa is a chronic inflammatory disease with multiple inflammatory nodules and abscesses. We aimed to compare split thickness skin graft (STSG) and flaps in bilateral chronic refractory axillary hidradenitis suppurativa.

**METHODS:**

Thirty patients were investigated from March 21, 2010 to March 20, 2015. Debridement of involved skin and subcutaneous fat was done until deep fascia. The second operation was a reconstructive procedure to cover bilateral axillary wounds with STSG in left side and random fasciocutaneous flaps in the right side.

**RESULTS:**

Mean age of patients was 35.2±9.3 years. There were 16 men (53.3%) and 14 women (46.7%). Duration of the disease before trial was 6.5±2.1 years. The association between pain at one-month follow-up for graft or flap sites was not significant. The patients did not have pain at flap and graft sites at three-month, six-month and one-year follow-ups. Twenty-four patients (80.0%) had normal ranges of motion at one-month follow-up. At six-month and one-year follow-ups, all patients had bilateral normal ranges of motion. All patients were satisfied from symmetry of flap and graft sites at six-month and one-year follow-ups. All patients were satisfied from graft and flap donor sites at six-month and one-year follow-ups. At one-month, three-month, six-month and one-year follow-ups, recurrence of hidradenitis suppurativa was not seen.

**CONCLUSION:**

Both STSGs and fasciocutaneous flaps were successful and satisfactory for tissue coverage in patients with axillary hidradenitis suppurativa. We recommend this technique in cases of bilateral axillary hidradenitis suppurativa.

## INTRODUCTION

Hidradenitis suppurativa is a chronic inflammatory disease with multiple inflammatory nodules and abscesses.^1^ The diagnosis is clinical and it usually involves axillary, perineal and inguinal regions.^2^^,^^3^ Early presentations of hidradenitis suppurativa respond to topical and systemic antibiotics. However, advanced and chronic stages require surgical intervention and debridement.^4^ Other treatment modalities include antiandrogens, anti-inflammatory drugs, radiotherapy, radiofrequency ablation and CO_2_ laser therapy.^5^

Surgical management of hidradenitis suppurativa is complex and requires a multidisciplinary approach.^6^^,^^7^ Refractory cases require surgical excision of involved tissues and wide debridement. Primary closure after debridement is not recommended, because of high recurrence rates. Loco-regional flaps, split thickness skin graft (STSG) and healing by secondary intention are three surgical reconstructive modalities to cover the debrided areas after granulation tissue appears.^4^ Skin grafting recovers fasters; however, secondary intention has less pain compared to STSG, because of donor site pain in grafting.^8^

Moderate and severe cases of hidradenitis suppurativa are accompanied by extensive inflammation and sinus tract formation. Thus, surgery is required in moderate to severe cases especially in chronic patients, who do not respond to non-surgical modalities.^9^ The modality of choice for tissue coverage of debrided areas in axillary hidradenitis suppurativa is controversial. STSG, flaps and secondary intention have been proposed, but none has shown superiority.^4^^,^^8^ Herein, we aimed to compare the results of STSG and flaps in cases of chronic axillary hidradenitis suppurativa. 

## MATERIALS AND METHODS

In a clinical trial, patients with bilateral hidradenitis suppurativa entered the study during March 21, 2010 to March 20, 2015. Inclusion criteria were age more than 18 years, age less than 70 years, diagnosis of bilateral axillary hidradenitis suppurativa, American Society of Anesthesiology classes of I and II and having informed consent to enter the study. Exclusion criteria were age less than 18 years, age more than 70 years, having unilateral axillary hidradenitis suppurativa and American Society of Anesthesiology classes of III, IV or V. 

**Fig. 1:**
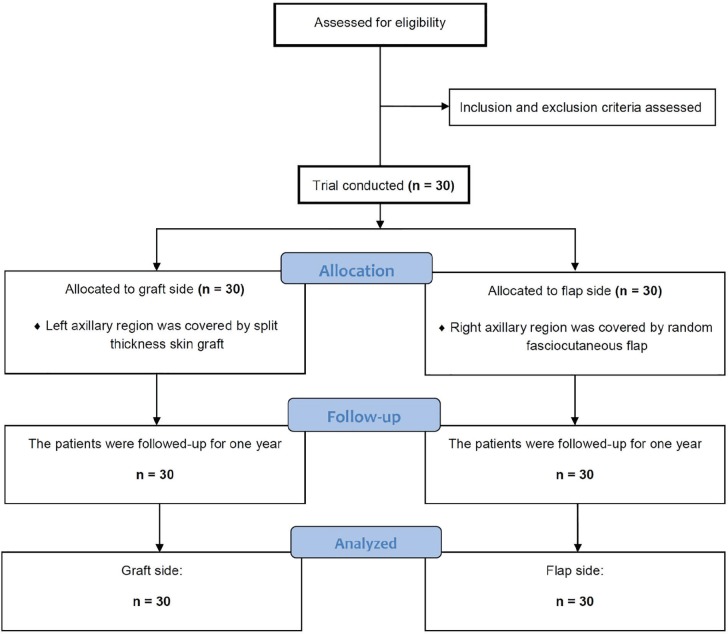
Consolidated standards of reporting trials for the study sample

The entire patients were followed up to one year. Figure 1 illustrated the study flow chart. Thirty patients met the inclusion and exclusion criteria and entered the study (Figure 1). All patients were scheduled to debridement for their first operation. Wide excision of axillary hidradenitis was conducted. Debridement of involved skin and subcutaneous fat was done until deep fascia. Then, a wet to dry depressing was applied. Irrigation and repeat dressings was used for two to three weeks. The patients were scheduled for their second operation after creation of appropriate granulation tissue. 

The second operation was a reconstructive procedure to cover bilateral axillary wounds with STSG and flaps. An appropriate size of STSG was harvested from anterior of right thigh in all patients. This graft was used to cover left axillary region. Right axillary wound was covered by random fasciocutaneous flap taken from para-scapular region. The place of taking flap was repaired primarily. Dressings of the graft site were uncovered after five to six days and dressings of the flap sites were uncovered on the postoperative day one. The patients were discharged on second postoperative days. 

Cefixime (400 mg per day) was administered for three days. The study was a clinical trial. The background variables were collected for the study included age, sex, body mass index (BMI) and past medical histories. The patients were scheduled for the operations and left and right sides were assessed and compared. The left side was graft side and the right side was flap side. The patients were followed-up for one year. They were visited on a regular basis one, three, six and twelve months after their second operation. 

They were assessed for complete recovery of the sides of graft and flap, recurrence, patients’ satisfaction, symmetry and cosmetics, range of motion of the shoulder, return to work, pain on flap or graft side, pain on flap or graft donor region and overall successfulness of the procedure. The successfulness of the procedure was defined as presence of necrosis of graft or flap not more than 25% of the covered area in the axillary region. Pain was assessed by Universal Pain Assessment Tool (UPAT). In UPAT, scores were 0 to 10, where 0 was no pain, 2 was mild pain, 4 to 6 were moderate pain and 8 and 10 were severe pain and worst pain possible respectively. 

The tool was illustrated to patients by visual surrogates and the data was submitted to checklist of the patients. All data of patients were submitted to a checklist and were analyzed. Statistical analysis was conducted by SPSS software (Version 21.0, Chicago, IL, USA). Variables were described by mean±standard variation (SD) and frequency (%). In the quantitative variables, we used the Student *t* test for normally distributed data and the Mann–Whitney *U* test for non-normally distributed data. We studied the association between qualitative variables using the χ^2^ test or Fisher’s exact test. 

P values less than 0.05 was considered statistically significant. Informed consent was obtained from all study patients. The protocol of this study was approved by research deputy of Faculty of Medicine, and Research Vice-Chancellor office of Shahid Beheshti University of Medical Sciences, Tehran, Iran. All consents were obtained by surgical residents and attending physicians. The protocol of this study was registered to Iranian Registry of Clinical Trial and approved by the number IRCT-M-233 It was also submitted to Shahid Beheshti University of Medical Sciences, Tehran, Iran by the number M-233.

## RESULTS

Thirty patients entered the study. Mean age of patients was 35.2±9.3 years old. There were 16 men (53.3%) and 14 women (46.7%). Table 1 illustrates background characteristics of the study patients. Duration of the disease before trial was 6.5±2.1 years. The patients received antibiotics during this period. Table 2 illustrates the type of antibiotics used by the study patients during this period. In addition, Table 3 tabulates hidradenitis suppurativa in anatomical locations other than axillae and previous non-pharmacological treatments in the study patients. All study patients had history of incision and drainage before trial. One patient had received radiotherapy.

**Table 1:** Background characteristics of the study patients

**Variable**	Mean±SD* or Frequency (%)
Age Sex Body Mass Index (BMI)	35.2±9.3 years 16 men (53.3) and 14 women (46.7) 28.1±5.5 kg/m^2^ Min=19.3 kg/m^2^; Max=39.1 kg/m^2^
Past history Hypertension Diabetes Mitral valve replacement	5 patients (16.7) 2 patients (6.7) 2 patients (6.7) 1 patient (3.3)
Duration of disease before surgery	6.5±2.1 years Min=3.0 years; Max=10.0 years
Follow-up	3.5±0.9 years Min=2.0 years; Max=5.0 years
Interval between excision and reconstruction	15.0±11.0 days Min=10.0 days; Max=20.0 days

*SD: Standard deviation

**Table 2:** Previous antibiotic use in study patients

**Antibiotic**	**Frequency (%)**
Cephalosporin or macrolide or fluoroquinolone Trimethoprim/Sulfamethoxazole Cephalosporin+Fluoroquinolone Cephalosporin+Macrolide Vancomycin+Macrolide Cephalosporin+Fluoroquinolone+Corticosteroid Fluoroquinolones+Vancomycin Cephalosporin+Macrolide+Corticosteroid Cephalosporin or Macrolide or Fluoroquinolone+Methotrexate Cephalosporin or Macrolide or Fluoroquinolone +Anticoagulant Cephalosporin+Vancomycin+Macrolide Cephalosporin+Fluoroquinolone+Macrolide Trimethoprim/Sulfamethoxazole+Methotrexate Vancomycin+Methotrexate	12 (40.0) 1 (3.3) 2 (6.7) 2 (6.7) 1 (3.3) 2 (6.7) 2 (6.7) 1 (3.3) 1 (3.3) 1 (3.3) 2 (6.7) 1 (3.3) 1 (3.3) 1 (3.3)
	

**Table 3:** Hidradenitis suppurativa in other anatomical locations and previous non-pharmacological treatments in the study patients

**Anatomical locations other than axillary region**	**Frequency (%)**
Perineum and perianal region Perineum and inguinal region	3 patients (10.0) 2 patients (6.7)
Perineum, perianal and inguinal region	3 patients (10.0)
Total	8 patients (26.7)
**Non-pharmacological treatments before the trial**	
Incision and drainage	29 patients (96.7)
Incision and drainage+radiotherapy	1 patient (3.3)

At one-month follow-up, two patients (6.7%) had mild pain at both flap and graft sites. One patient (3.3%) had mild pain only at flap site. One patient (3.3%) had moderate pain only at flap site. Two patients (6.7%) experienced mild pain only at the graft site. Two patients (6.7%) suffered from moderate pain only at graft site. Twenty-two patients (73.3%) reported pain neither at the flap site nor in the graft site. The association between pain at one-month follow-up for graft or flap sites was not significant (*p*>0.05). 

The patients did not have pain at flap and graft sites at three-month, six-month and one-year follow-ups. At one-month follow-up, one patient (3.3%) had limited range of motion at right (flap site) and left (graft site) shoulders. One patient (3.3%) reported limited range of motion only at left (graft side) shoulder. Three patients (10.0%) experienced limited range of motion only at right (flap site) shoulder. Twenty-four patients (80.0%) demonstrated normal ranges of motion (*p*>0.05). At three-month follow-up, only one patient (3.3%) revealed limited range of motion at right (flap site) shoulder. 

At six-month and one-year follow-ups, all patients illustrated bilateral normal ranges of motion. At one-month follow-up, five patients (16.7%) were satisfied from symmetry of flap, but were not satisfied from symmetry of graft. Four patients (13.3%) reported satisfaction from symmetry of graft, but were not satisfied from symmetry of flap. Twenty-one patients (70.0%) were satisfied from symmetry of both flap and graft sites (p > 0.05). At three-month follow-up, two patients (6.7%) depicted satisfaction from symmetry of flap, but were not satisfied from symmetry of graft. 

One patient (3.3%) was found satisfied from symmetry of graft, but was not satisfied from symmetry of flap. Twenty-six patients (86.7%) revealed satisfaction from symmetry of both flap and graft sites (*p*>0.05). All patients were satisfied from symmetry of flap and graft sites at six-month and one-year follow-ups. At one-month follow-up, two patients (6.7%) had more than 25% necrosis on flaps and two patients (6.7%) had more than 25% necrosis on grafts. Twenty-six patients (86.7%) did not show any necrosis (*p*>0.05). 

Additional necrosis did not occur at three-month, six-month and one-year follow-up visits. At one-month follow-up, 7 patients (23.3%) were absent at their works. All patients attended their works in three-month, six-month and one-year follow-ups. At one-month follow-up, all patients reported satisfaction from flap donor sites. Six patients (20.0%) had mild to moderate pain at graft donor site. Twenty-four patients (80.0%) were satisfied from graft donor sites (*p*>0.05). The same findings were found at three-month follow-up. All patients were satisfied from graft and flap donor sites at six-month and one-year follow-ups. At one-month, three-month, six-month and one-year follow-ups, recurrence of hidradenitis suppurativa was not observed in the studied patients.

## DISCUSSION

The present study revealed that both STSG and para-scapular flaps were safe and useful techniques for reconstruction of debrided sites in chronic hidradenitis suppurativa of the axillary region. Eight patients had pain on their graft or flap sites at one-month follow-up, but none of the methods was superior. The patients did not have any pain on later follow-ups. Range of motion of shoulders was also normal at six-month and one-year follow-ups. Symmetry of axillae was favorable in both graft and flap sites. Only four patients had more than 25% necrosis on one-month follow-up.

It is essential that tissue coverage be used for moderate to severe chronic hidradenitis suppurativa.^8^^,^^9^ Antibiotics and other modalities are usually used before surgical reconstructions.^4^ All patients in our study used antibiotics. Incision and drainage were also conducted in all patients. We scheduled our patients to a two-stage surgery. All patients had bilateral axillary hidradenitis suppurativa. We compared two sides of patients, while left sides were covered by STSGs and right sides were covered by flaps. Results of both sides were satisfactory. This study had limited bias and confounding, because case and controls were the same group of patients and the comparison was done between two sides of the patients.

It is recommended that skin grafts, flaps and secondary healing are three potential surgical modalities to reconstruct the axilla after wide debridement and multiple irrigations. However, none of these modalities are superior to others.^4^ Secondary healing seems to have less pain that grafting.^8^ Wormald *et al.* conducted a prospective study to compare the outcomes of thoracodorsal artery perforator (TDAP) flap and STSG in the management of chronic axillary hidradenitis suppurativa. They concluded that both methods improved quality of life. However, their results revealed that TDAP flaps had greater benefits regarding quality of life, recovery, rate of complications and number, regarding overall procedures.^10^

Elgohary *et al.* reported that regarding TDAP flaps on moderate to severe axillary hidradenitis suppurativa, the patients had good cosmetic and functional results with 100% success rate.^11^ Busnardo *et al.* used TDAP flaps for 12 patients with severe hidradenitis suppurativa after radical excision and showed a good range of motion and increased arm abduction. In addition, they demonstrated that flap had advantages compared to other tissue coverages including proximity to axilla and quality and thickness of skin.^12^

Our study was designed to compare STSG and para-scapular flap in a group of patients with bilateral axillary hidradenitis suppurativa. Both methods were used in all patients. The results were satisfactory and both techniques were successful for quality of life, return to work and range of motion of shoulder. Pain and flap necrosis were minimal. Elboraey *et al. *reported results of six patients with localized axillary hidradenitis suppurativa. They performed radical excision of localized axillary hidradenitis suppurativa with immediate or delayed perforator-based propeller flap defect closure.^13^

Different flap techniques have been used by other investigators to cover the excised site at axilla in patients with chronic axillary hidradenitis. Marchesi *et al.* used immediate reconstruction with 15 local thoracodorsal artery perforator flaps and 2 muscle-sparing latissimus dorsi flaps.^14^ Chuang *et al.* applied eight versatile transpositional fasciocutaneous flap in seven patients and observed satisfactory results.^15^ Rehman *et al.* used TDAP V-Y advancement flaps (type I) to close in a single-stage procedure in four patients and their results were satisfactory.^16^ Hallock used island thoracodorsal artery perforator V-Y advancement flap.^17^

We used graft and flap to reconstruct debrided areas in axillary region. Left axillary region was covered by graft and right axillary region was covered by para-scapular flap. Both coverages were successful and satisfactory outcomes were obtained. We conducted a two-stage operation. First, excision and wide debridement of the hidradenitis was undertaken. Second operation was carried out two to three weeks later to reconstruct the affected area by graft or flap (15.0±10.0 days). Regular irrigation and wet to dry dressings were applied during this interval.

A number of authors have used flaps for tissue coverage of axillary region after debridemen.^13^^-^^17^ We used both flap and STSG. Mohos *et al.* applied thoracodorsal fasciocutaneous flaps to cover axilla in 14 patients with hidradenitis suppurativa. Two of these patients had bilateral involvement. They illustrated that flap coverage was an ideal solution for tissue coverage in chronic axillary hidradenitis suppurativa.^18^ All our patients also had bilateral involvement. Graft and flap coverages were successful in our investigation. Calibre *et al.* used meshed STSG to cover axillary region in five patients with hidradenitis suppurativa. They performed the procedure in a single stage and reported fast healing and good quality and patients’ comfort.^19^

We did not find any significant difference between outcomes of graft and flap coverage. Shor-term differences were not statistically significant. We used staged technique for graft and flap coverage. Pearce and Richardson conducted a staged procedure in seven patients with axillary hidradenitis suppurativa. They reported an average of ten days of hospital admission with potential health system costs. They used STSG for tissue coverage.^20^ We also used staged technique. We assumed that an interval of regular irrigation and wet to dry dressings could minimize graft and flap failures. However, hospital stays and costs should be considered, while staged procedures are used for reconstruction.

Our study was unique in respect to high number of patients with bilateral chronic axillary hidradenitis suppurativa. In addition, we used STSG and flap in all study patients. Patients and investigators compared flap and graft in each patient between two sides and bias and confounding variables were minimal. Pain was minimal at follow-ups. Range of motion and symmetry of axillar was optimal. Quality of life and return to work were satisfactory. In conclusion, both STSGs and fasciocutaneous flaps were successful and satisfactory for tissue coverage in patients with axillary hidradenitis suppurativa. We recommend this technique in cases of bilateral axillary hidradenitis suppurativa. 
